# Organellar Gene Expression and Acclimation of Plants to Environmental Stress

**DOI:** 10.3389/fpls.2017.00387

**Published:** 2017-03-21

**Authors:** Dario Leister, Liangsheng Wang, Tatjana Kleine

**Affiliations:** Plant Molecular Biology, Department Biology I, Ludwig-Maximilians-Universität MünchenPlanegg-Martinsried, Germany

**Keywords:** organellar gene expression, plastid, retrograde signaling, *Arabidopsis*, acclimation

## Abstract

Organelles produce ATP and a variety of vital metabolites, and are indispensable for plant development. While most of their original gene complements have been transferred to the nucleus in the course of evolution, they retain their own genomes and gene-expression machineries. Hence, organellar function requires tight coordination between organellar gene expression (OGE) and nuclear gene expression (NGE). OGE requires various nucleus-encoded proteins that regulate transcription, splicing, trimming, editing, and translation of organellar RNAs, which necessitates nucleus-to-organelle (anterograde) communication. Conversely, changes in OGE trigger retrograde signaling that modulates NGE in accordance with the current status of the organelle. Changes in OGE occur naturally in response to developmental and environmental changes, and can be artificially induced by inhibitors such as lincomycin or mutations that perturb OGE. Focusing on the model plant *Arabidopsis thaliana* and its plastids, we review here recent findings which suggest that perturbations of OGE homeostasis regularly result in the activation of acclimation and tolerance responses, presumably via retrograde signaling.

## Introduction: The Plastid Gene-Expression Machinery is of Mixed Genetic Origin

**FIGURE 1:**
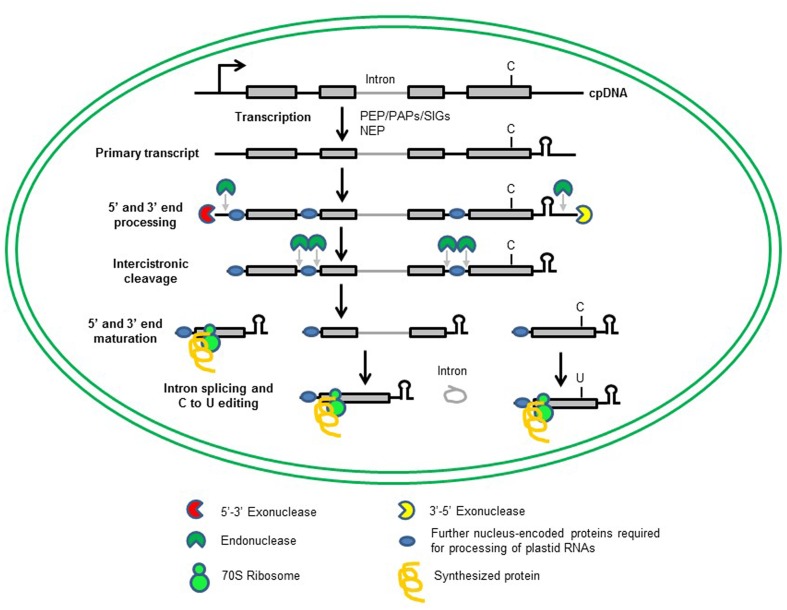
**Transcription of chloroplast genes and maturation of chloroplast RNAs**. Most of the chloroplast genes are organized in operons, and are transcribed as polycistronic RNAs from single promoters (bent arrow). Transcription of chloroplast mRNA depends on two types of RNA polymerases, a plastid-encoded PEP and one or two nucleus-encoded NEPs. The primary transcript undergoes several steps of maturation that include 5′ and 3′ end processing, intercistronic cleavage, 5′ and 3′ end maturation, intron splicing and RNA editing to produce functional RNAs. For these events to take place, a whole series of nucleus-encoded proteins are needed (blue oval or segmented circles). Mature plastid RNAs are translated by bacterial-type 70S ribosomes using the set of tRNAs encoded by the plastid genome.

Like mitochondria, plastids – as descendants of cyanobacterium-like progenitors – are of endosymbiotic origin (Raven and Allen, 2003). During evolution, plastids have lost most of their genes to the nucleus, and the plastid genomes of embryophytes contain only 90 to 100 genes (Wicke et al., 2011). However, plastids contain 3000–4000 proteins which function in photosynthesis, the biosynthesis of fatty acids, amino acids, hormones, vitamins, nucleotides, and secondary metabolites, and intracellular signaling (Leister and Kleine, 2008). Thus, plastids encode only a small fraction of the proteins needed to sustain the processes they host. The relatively few genes remaining in the organelles code for proteins involved in plastid gene expression (PGE) or energy production. But while its gene complement is small, PGE is a very complex process. This is because plastids have retained a prokaryotic gene-expression apparatus which is combined with eukaryotic inventions, and its polycistronic transcripts must undergo numerous post-transcriptional maturation steps (**Figure 1**). In higher plants, plastid transcription is performed by three different RNA polymerases: two monomeric, nucleus-encoded (NEP) RNA polymerases and a plastid-encoded (PEP) *E. coli*-like enzyme (Lerbs-Mache, 2011; Börner et al., 2015). Moreover, the multisubunit enzyme requires a set of polymerase-associated proteins (PAPs) and sigma factors (SIGs) for function, which are themselves encoded in the nucleus (Lerbs-Mache, 2011; Börner et al., 2015; Chi et al., 2015).

The polycistronic RNAs synthesized by plastid polymerases require extensive processing, including 5′ and 3′ trimming, intercistronic cleavage, splicing and editing, for which a plethora of nucleus-encoded proteins are needed (**Figure 1**; del Campo, 2009; Stern et al., 2010; Hammani et al., 2014; Kleine and Leister, 2015; Schmitz-Linneweber et al., 2015). Plastid proteins are synthesized by bacterial-type 70S ribosomes using a set of tRNAs that is entirely encoded in the plastid genome (Tiller and Bock, 2014; Sun and Zerges, 2015). The plastid ribosome itself consists of the large (50S) and small (30S) multi-component ribosomal subunits, each comprising one or more plastid-encoded ribosomal RNA species (rRNAs), and furthermore, plastid- and nuclear-encoded proteins (Yamaguchi and Subramanian, 2000; Yamaguchi et al., 2000).

## Impact of Environmental Changes on the Pge Machinery

Plastid gene expression is crucial for plant development and photosynthesis, and must therefore respond appropriately to developmental and environmental changes. It does so, in part, by modifying transcription levels. Thus, the hormone abscisic acid (ABA) represses the transcription of plastid genes (Yamburenko et al., 2013), and also the circadian clock (Noordally et al., 2013), light, temperature and plastid development differentially modulate transcription in the plastid (reviewed in: Börner et al., 2015). Recently, it was proposed that light-related plastid transcriptional responses are integrated by especially SIG5 (Belbin et al., 2017). In detail, the transcriptional response to light intensity, as well as the response to the relative proportions of red and far red light through phytochrome and photosynthetic signals, and the circadian regulation of plastid transcription (which is predominantly dependent on blue light and cryptochrome), are regulated by SIG5 (Belbin et al., 2017). In bacteria, responses to stress rely mainly on phosphorylation-dependent signal transduction systems, which act upon transcriptional regulons either by activating DNA-binding two-component response regulators or sigma factors (Marles-Wright and Lewis, 2007). The example of the involvement of SIG5 in several light-dependent pathways (Belbin et al., 2017) and that in general, SIG5 and SIG6 are involved in multiple signaling pathways, suggest that this type of regulation may also be important in plants (reviewed in: Chi et al., 2015). Transcription rates of plastid genes have been shown to be modulated by electron-transfer inhibitors and whether incident light preferentially excites photosystem I or photosystem II (Pfannschmidt et al., 1999). Another PEP-associated protein is the plastid transcription kinase PTK, which responds to changes in the thiol/disulfide redox state mediated by glutathione (Baginsky et al., 1999), and has been shown to target SIG6 (Schweer et al., 2010). *In organello* run-on transcription and phosphorylation assays indeed suggest that the regulation of plastid transcription under different light intensities depends on both glutathione and phosphorylation status (Baena-Gonzalez et al., 2001).

Cluster analyses of plastid transcriptomes from mutants with severe photosynthetic defects or from plants exposed to stresses suggest that the accumulation of specific plastid RNAs is regulated in response to the physiological state of the organelle (Cho et al., 2009). Because organellar multiprotein complexes – including many components of PGE and the photosynthetic machinery – typically contain both plastid- and nucleus-encoded subunits, tight coordination of the activity of the two compartments is necessary. A part of this takes place at the transcript level, as revealed by an analysis of co-regulation based on 1300 transcription profiles obtained under different environmental conditions and in different genetic backgrounds (Leister et al., 2011). The tightest co-regulation was generally observed for genes located in the same compartment. Strikingly however, under stress conditions, nucleus-plastid coregulation could predominate over intracompartmental networks, i.e., specific sets of nuclear and organellar photosynthesis genes were co-expressed. Moreover, when genes were ranked according to the number of situations in which their expression levels were altered by at least twofold (Leister et al., 2011), *NDHF* (the plastid gene for a subunit of NADH dehydrogenase) was classified as “very highly responsive,” as it reacted in 104 of 413 tested states. Several other plastid genes were highly responsive, showing that coordinated transcriptional regulation occurs on a broader scale. The relevance of transcriptional control in the plastid is underlined by changes in the expression of nucleus-encoded sigma factors (which mediate transcription initiation by PEP): *SIG1* and *SIG5* mRNA levels are regulated in 110 and 65 conditions, respectively (Leister et al., 2011) and other studies confirm that sigma factors respond to environmental conditions and are involved in acclimation processes (see above; summarized in: Börner et al., 2015; Chi et al., 2015). Indeed, SIG5 is considered as a multiple stress-responsive sigma factor (Nagashima et al., 2004; Chi et al., 2015), because *SIG5* is induced by exposure to high light, low temperature, high salt and high osmotic pressures (Nagashima et al., 2004), blue light (Tsunoyama et al., 2002), and ABA (Yamburenko et al., 2015).

Steady-state mRNA levels at any given time reflect the relationship between transcription rate and mRNA degradation rate. In bacteria, the latter plays an important role in controlling gene expression (Hui et al., 2014). Since sessile plant species cannot escape from unfavorable environmental conditions, it is conceivable that they have had to develop more flexible response mechanisms. Indeed, it is generally accepted that the control of PGE has shifted to post-transcriptional events over the course of evolution (Barkan and Goldschmidt-Clermont, 2000; Stern et al., 2010), especially in mature chloroplasts (Sun and Zerges, 2015). Thus, unlike redox regulation of transcription in mustard (Pfannschmidt et al., 1999) and ABA-mediated repression of transcriptional activity of chloroplast genes in barley (Yamburenko et al., 2013), levels of individual plastid mRNAs in spinach (Klaff and Gruissem, 1991) and barley (Kim et al., 1993) during plant development are mainly determined by alterations in stability, with half-lifes of many hours or even days – much more stable than bacterial mRNAs with typical lifetimes of seconds to hours (Radhakrishnan and Green, 2016). This suggests that the differential accumulation of chloroplast mRNAs – at least under these conditions – is primarily regulated at the post-transcriptional level. Consequently, RNA stability is probably the dominant factor governing mRNA levels in plastids. Interestingly, a genome-wide study of mRNA decay rates in *A. thaliana* cell cultures showed that nuclear transcripts encoding mitochondrial, chloroplast and peroxisomal proteins tend to have a high proportion of transcripts with long half-lifes (Narsai et al., 2007). This may be largely due to the fact that many of the proteins known to be located in these organelles are associated with intermediate metabolism and energy. Interestingly, transcripts encoding pentatricopeptide repeat (PPR) proteins, which have short half-lifes, are exceptions to this generalization (Narsai et al., 2007). The latter finding is corroborated by an analysis of mRNA half-life changes in response to cold stress in *Arabidopsis* (Chiba et al., 2013). When mRNA levels vary depending on developmental stage, environmental factors or intracellular signals, earlier processing events can be the main determining factor (Monde et al., 2000). PPR proteins are important here also, for they are mainly targeted to chloroplasts and/or mitochondria and, as RNA-binding proteins, they participate in RNA editing, splicing, stability, and translation (Barkan and Small, 2014).

## Organellar Gene Expression and Acclimation to Abiotic Stress Conditions

Many of the genes on which plastid and mitochondrial gene expression (organellar gene expression; OGE) depends reside in the nuclear genome, which provides for direct control of OGE by nuclear factors (via “anterograde signaling”). Conversely, organelles transmit information relating to their developmental and metabolic states to the nucleus (“retrograde signaling”), enabling nuclear gene expression to be modulated in accordance with their physiological needs (reviewed in: Kleine et al., 2009; Chi et al., 2013; Bobik and Burch-Smith, 2015; Kleine and Leister, 2016). Retrograde signals are presumed to originate from OGE itself, the tetrapyrrole pathway, the redox state of the organelles, levels of reactive oxygen species (ROS) in the organelles (such as singlet oxygen, hydrogen peroxide, superoxide anion radicals, and hydroxyl radicals) and metabolites [such as cyclocitral, 3′-phophoadenosine 5′-phosphate (PAP) and methylerythritol cyclodiphosphate (MecPP)] (reviewed in: Kleine et al., 2009; Terry and Smith, 2013; Bobik and Burch-Smith, 2015; Colombo et al., 2016; Dietz et al., 2016). Recent research extends the previous view of retrograde signaling to mainly affect transcriptional reprogramming to also include posttranslational control, which involves the ubiquitin-proteasome system (reviewed in: Woodson, 2016). ROS signatures and metabolite signals control acclimation processes involving the alteration of gene expression and translation which is reviewed elsewhere (Dietz et al., 2016; Kleine and Leister, 2016). Furthermore, a picture emerges in which considerable cross-talk between established signaling pathways takes place. As examples, retrograde signaling pathways converge with photoreceptor pathways (Martin et al., 2016), regulation of flowering time (Feng et al., 2016) and/or hormonal signaling cascades (reviewed in: Bobik and Burch-Smith, 2015; Gollan et al., 2015). In this review, we focus on the relationship between OGE and acclimation responses to abiotic stresses.

### The *gun* Mutants, ABA, and Abiotic Stresses

Treatment with inhibitors of OGE, such as chloramphenicol or lincomycin, or the carotenoid biosynthesis inhibitor norflurazon results in reduced expression of nuclear genes encoding plastid proteins (Oelmuller and Mohr, 1986). In the best-known screen for retrograde signaling mutants, *genomes uncoupled* (*gun*) seedlings were mutagenized and mutants that continued to express a nucleus-encoded plastid protein in the presence of norflurazon were selected (Susek et al., 1993). Recent research confirms that functioning chloroplasts are essential for plant acclimation to adverse environmental conditions (for an overview, see **Table 1**). Inactivation of the H-subunit of the plastid Mg-chelatase (GUN5) results in cold (4°C) sensitivity, and it was suggested that perturbation of plastid function in *gun5* mutants could result in inhibition of protein synthesis and impair plant performance at low temperatures (Kindgren et al., 2015). In this context, it is postulated that enhanced tetrapyrrole biosynthesis might confer drought tolerance via ROS detoxification (reviewed in: Nagahatenna et al., 2015).

**Table 1:** Phenotypes of *oge* mutants.

ATG number	Mutant name	Description	Localization	Mutant identification/ availability	Type of mutation	Mutant phenotype	Reference
AT2G31400	*gun1-1*	GENOMES UNCOUPLED 1, GUN1	Chloroplast	Screen of an EMS-mutagenized M2 population for mutants that still accumulate *LHCB1* mRNA when grown on norflurazon	Ala259Val	Defective in plastid-to-nucleus signaling, ABA tolerant	Koussevitzky et al., 2007; Cottage et al., 2010
AT3G59400	*gun4-1*	GENOMES UNCOUPLED 4, GUN4	Chloroplast	Screen of an EMS-mutagenized M2 population for mutants that still accumulate *LHCB1* mRNA when grown on norflurazon	Leu88Phe	Pale green and defective in plastid-to-nucleus signaling, ABA tolerant	Larkin et al., 2003; Voigt et al., 2010
AT5G13630	*gun5-1*	ABA-BINDING PROTEIN, ABAR, CCH, CCH1, CHLH, GENOMES UNCOUPLED 5, GUN5	Chloroplast	Screen of an EMS-mutagenized M2 population for mutants that still accumulate *LHCB1* mRNA when grown on norflurazon	Ala990Val	Defective in retrograde plastid-to-nucleus signaling, sensitive to low temperatures, ABA tolerant	Mochizuki et al., 2001; Voigt et al., 2010; Kindgren et al., 2015
AT1G56570	*pgn*	PENTATRICOPEPTIDE REPEAT PROTEIN FOR GERMINATION ON NACL, PGN	Mitochondrion	ABRC	SALK_141937, truncated protein	Hypersensitive to ABA, glucose, and salinity	Laluk et al., 2011
AT3G23700	*srrp1*	S1 RNA-BINDING RIBOSOMAL PROTEIN 1, SRRP1	Chloroplast	ABRC	SAIL 299 A11, T-DNA is inserted approximately 100 base pairs upstream of the start codon of SRRP1	Poorer seedling growth and less cotyledon greening on MS medium supplemented with ABA	Gu et al., 2015
AT4G17040	*hon5*	CLP PROTEASE R SUBUNIT 4, CLPR4, HAPPY ON NORFLURAZON 5, HON5	Chloroplast	Screen of an EMS-mutagenized M2 population for mutants that were able to green when grown on a low dose of norflurazon under dim light	G to A exchange at the 3’ end of first intron	Mutants are green in the presence of norflurazon and plastid protein homeostasis is disturbed	Saini et al., 2011
AT5G13650	*hon23*	SUPPRESSOR OF VARIEGATION 3, SVR3, HAPPY ON NORFLURAZON 23, HON23	Chloroplast	Screen of an EMS-mutagenized M2 population for mutants that were able to green when grown on a low dose of norflurazon under dim light	Arg438His	Mutants are green in the presence of norflurazon and plastid protein homeostasis is disturbed	Saini et al., 2011
AT2G36990	*soldat8*	RNAPOLYMERASE SIGMA-SUBUNIT F, SIG6, SIGF, SIGMA FACTOR 6, SOLDAT8	Chloroplast	Screen for second-site mutations of *fluorescent (flu)* that attenuate the *flu* phenotype	Gln354Stop	Accumulates reduced amounts of chlorophyll and delays chloroplast development, high light tolerant	Coll et al., 2009
At2g03050	*soldat10*	mTERF1, EMB93, EMBRYO DEFECTIVE 93, SINGLET OXYGEN-LINKED DEATH ACTIVATOR 10, SOLDAT10	Chloroplast	Screen for second-site mutations of *fluorescent (flu)* that attenuate the *flu* phenotype	Pro54Leu	Suppresses ^1^O_2_-induced cell death, high light tolerant	Meskauskiene et al., 2009
AT4G14605	*mda1*	mTERF5	Chloroplast	Sequence similarity searches in Arabidopsis genome databases using Arabidopsis RUG2 protein as a query	*mda1-1*: SALK_097243, truncated protein lacking 2 mTERF motifs; *mda1-2*: SAIL_425_E03, truncated protein lacking 5 mTERF motifs	Altered chloroplast morphology and plant growth, reduced pigmentation of cotyledons, leaves, stems and sepals, salt and osmotic stress tolerant.	Robles et al., 2012
AT5G55580	*mterf9*	mTREF9	Chloroplast	Reverse genetics	*mterf9*: N857510, truncated protein lacking 2 mTERF motifs. *twr-1*: Q467Stop	Pale, stunted growth, and reduced mesophyll cell numbers, altered responses to sugars, ABA, salt and osmotic stresses	Robles et al., 2015
AT4G02990	*rug2-1*	mTERF4	Chloroplast, mitochondrion	Screen of an EMS-mutagenized M2 population for mutants affecting leaf morphology	Pro420Leu	Reduced growth, leaves with green and white sectors, altered chloroplast and mitochondrion development, hypersensitive to temperature stress	Quesada et al., 2011
At3g60400	*shot1*	mTERF18	Mitochondrion	Suppressor of *hot1-4* (a dominant-negative allele of *HSP101*)	*shot1-1*: Gly_105_/Asp; *shot1-2*: premature stop codon, truncated protein.	Short hypocotyl in the dark, growth reduction, less oxidative damage, suppresses other heat-sensitive mutants, heat tolerant	Kim et al., 2012
AT5G30510	*rps1*	RIBOSOMAL PROTEIN S1, RPS1	Chloroplast	ABRC	*rps1*: CS874869, T-DNA is inserted 6-bp upstream of the 5’-untranslated region of the *RPS1* gene	Perturbation of HSF-mediated heat stress response, loss of heat tolerance	Yu et al., 2012
AT2G33800	*rps5*	RIBOSOMAL PROTEIN S5, RPS5	Chloroplast	Screen of an EMS-mutagenized M2 population for mutants with abnormal leaf color	Gly180Glu	Pale yellow inner leaves, reduced growth, reduced abundance of chloroplast 16S rRNA, hypersensitive to cold stress	Zhang et al., 2016
AT3G20930	*orrm1*	ORGANELLE RRM PROTEIN 1, ORRM1	Chloroplast	ABRC	SALK_072648, T-DNA is inserted in first exon	Developmental delay and pale green inner leaves when grown at 4°C	Sun et al., 2013; Wang et al., 2016
At3g53460	*cp29a*	CHLOROPLAST RNA-BINDING PROTEIN 29, CP29	Chloroplast	Obtained from ABRC and GABI-Kat	*cp29a-1*: SALK_003066, T-DNA is inserted in third exon; *cp29a-6*: 001G06, T-DNA is inserted in second intron	Developmental delay and pale green leaves when grown at 8°C	Kupsch et al., 2012
AT4G24770	*cp31a*	31-KDA RNA BINDING PROTEIN, CP31, RBP31	Chloroplast	ABRC	*cp31a-1*: SALK_109613, T-DNA is inserted in third exon; *cp31a-3*: SAIL_258H02, T-DNA is inserted in first exon	Developmental delay and pale green leaves when grown at 8°C	Tillich et al., 2009
AT1G70200	*rbd1*	HIGH PHOTOSYNTHETIC EFFICIENCY 1, HPE1, RBD1	Chloroplast	ABRC	*rbd1-1*: SALK_041100, T-DNA is inserted in third exon; *rbd1-2*: SALK_012657, T-DNA is inserted in first exon	Pale green inner leaves when grown at 4°C	Wang et al., 2016
AT4G39040	*cfm4*	CRM FAMILY MEMBER SUBFAMILY 4, CFM4	Chloroplast	ABRC	*cfm4-1*: SALK_076439, T-DNA is inserted in third exon; SALK_126978, T-DNA is inserted in third exon	Retarded seed germination and growth under stress conditions	Lee et al., 2014
AT5G26742	*rh3*	ATRH3, EMB1138, EMBRYO DEFECTIVE 1138, RH3	Chloroplast	ABRC	*rh3-4*: SALK_005920, T-DNA is inserted in ninth intron	Retarded growth phenotype and defects in chloroplast biogenesis and photosynthetic activity	Gu et al., 2014

A recurring feature of *oge* mutants is their atypical response to ABA. The tetrapyrrole biosynthesis proteins GUN4 and GUN5 (Voigt et al., 2010) and the plastid-targeted PPR protein GUN1 (Cottage et al., 2010) enhance seedling development in the presence of ABA. On the other hand, loss of the mitochondrial PPR protein PENTATRICOPEPTIDE REPEAT PROTEIN FOR GERMINATION ON NaCl (PGN) results in hypersensitivity to ABA, glucose, and salinity (Laluk et al., 2011). It was suggested that *pgn* plants accumulate large amounts of ABA, and transcripts of ABA-related genes, as well as mitochondrial transcripts, are up-regulated. Levels of *ABI4* and *ALTERNATIVE OXIDASE1a* mRNAs, whose products are known for their roles in mitochondrial retrograde signaling, are particularly affected (Laluk et al., 2011). Thus, PGN is assumed to help neutralize ROS in mitochondria during abiotic and biotic stress responses, probably via retrograde signaling. Another mutant with perturbed RNA metabolism, *srrp1* (*S1 RNA-binding ribosomal protein 1*), in which intron splicing of plastid *trnL* and processing of 5S rRNA were altered, does not display any visible phenotype under normal growth conditions, but seedling development is impaired in the presence of ABA (Gu et al., 2015). Furthermore, mutants lacking WHIRLY1 were shown to be less sensitive to salicylic acid and ABA during germination (Isemer et al., 2012a). The DNA-binding protein WHIRLY1 can translocate from plastids to the nucleus, making it one of the most promising candidate mediators of signaling between organelles and the nucleus (Isemer et al., 2012b). WHIRLY1 was recently proposed to serve as a redox sensor in plastid-to-nucleus retrograde signaling and to mediate cross tolerance, including acclimation responses (Foyer et al., 2014). Finally, application of ABA can partially restore mRNA expression of the nucleus-encoded plastid protein Lhcb1.2 in NF-treated wild-type plants, supporting the view that OGE and ABA signaling are interconnected (Voigt et al., 2010). Indeed, the transcription factor ABSCISIC ACID INSENSITIVE4 (ABI4) which has emerged as a central player in many signaling processes during plant development (reviewed in: Léon et al., 2012), has been directly associated with retrograde signaling (Koussevitzky et al., 2007; Giraud et al., 2009). Interestingly, it was shown more than a quarter of a century ago that, while *abi* mutations had no apparent effect on freezing tolerance, cold-acclimated ABA biosynthesis (*aba*) mutants were markedly impaired in freezing tolerance (Gilmour and Thomashow, 1991), indicating that ABA levels can affect freezing tolerance. In addition, temporal and spatial interactions of ABA with ROS signals were shown to play a key role in the regulation of systemic acquired acclimation of plants to heat stress (Suzuki et al., 2013) and ABA is required for plant acclimation to a combination of salt and heat stress (Suzuki et al., 2016). The manifold links between ABA and acclimation responses, and the ABA phenotypes of *oge* mutants, imply that a functional OGE system is essential for proper acclimation responses.

### The *hon*, *soldat*, and *mterf* Mutants in the Context of Abiotic Stresses

Treatments with synthetic inhibitors expose plants to highly artificial conditions and are only effective if applied at an early stage of seedling development (Oelmuller and Mohr, 1986). To approximate physiological conditions more closely, a screen was designed that used a reduced concentration of norflurazon and low light levels. This resulted in the identification of *happy on norflurazon* (*hon*) mutants, which remain green in the presence of a low dose of norflurazon (Saini et al., 2011). Because some *hon* mutations were mapped to genes coding for a subunit of the plastid-localized Clp protease complex (*ClpR4* = *HON5*) and a putative plastid translation elongation factor (*HON23*), *hon* mutations can clearly interfere with PGE (Saini et al., 2011). Interestingly, *hon* seedlings were more resistant than WT to simultaneous exposure to low temperature and high light (Saini et al., 2011). The *soldat8* and *soldat10* (singlet oxygen-linked death activator) seedlings identified in an earlier screen for second-site mutations that suppress the singlet oxygen (^1^O_2_)-mediated stress response of *fluorescent* (*flu*) seedlings (Coll et al., 2009; Meskauskiene et al., 2009) behave similarly. The two *soldat* lines are also mutated in genes for proteins related to PGE: *soldat8* is mutant for *SIG6* (see above; Coll et al., 2009) and *soldat10* is defective in the gene encoding mitochondrial Transcription Termination Factor1 (mTERF1) (Meskauskiene et al., 2009), thus linking PGE to the ^1^O_2_-mediated cell-death responses.

Most members of the mTERF family, which are found in metazoans (four each in human and mouse) and plants (35 in *A. thaliana*), are located in mitochondria and/or plastids, where they regulate OGE at different steps of transcription or translation (Kleine and Leister, 2015). Moreover, several *mterf* mutants have been linked to stress responses. Thus *mda1* (*mterf5*) and *mterf9* seedlings are less susceptible to salt and osmotic stresses, perhaps owing to reduced sensitivity to ABA (Robles et al., 2012, 2015). The *rug2-1* (*mterf4*) mutant is sensitive to temperature stress. When grown at 26°C, *rug2-1* growth is arrested, whereas at 16°C its mutant phenotype is fully suppressed (Quesada et al., 2011). The concept of ROS as retrograde signals in heat stress responses has been reviewed elsewhere (Sun and Guo, 2016). An example for a perturbation in OGE homeostasis contributing to enhanced thermotolerance is the *mterf18*/*shot1* mutant (Kim et al., 2012). The mitochondrial mTERF18/SHOT1 protein was identified as a suppressor of *hot1-4* (a dominant-negative allele of HSP101) (Kim et al., 2012). The increase in thermotolerance (after heat acclimation at 38°C for 90 min, followed by 2 h at 22°C, then heat-shocked at 45°C for several hours) in the *shot 1* mutant is associated with the accumulation of lower amounts of ROS, and thus a higher tolerance of oxidative stress. Moreover, the plastid ribosomal protein S1 (RPS1) is induced after 2 h of heat treatment (38°C) in the dark, and down-regulation of RPS1 has been shown to severely impair the heat stress-activated expression of *HsfA2* and its target genes, resulting in a loss of heat tolerance (Yu et al., 2012). Interestingly, the level of RPS1 is controlled by GUN1 (Tadini et al., 2016).

### PGE and Chilling Tolerance

Plastid gene expression is also important for chilling (low but not freezing temperatures; 4–12°C) tolerance, as exemplified by the *rps5* mutant identified in a screen for genes required for plastid development. The missense mutation in plastid ribosomal protein S5 reduces growth rate and inner leaves remain pale yellow. Furthermore, a variety of photosystem I and II proteins, as well as plastid ribosomal proteins are underrepresented. Levels of proteins associated with stress responses to cold stress are decreased in *rps5*, and overexpression of plastid *RPS5* improves tolerance to cold (Zhang et al., 2016). In a systematic screen of 11,000 T-DNA *A. thaliana* insertion mutants for genes involved in chilling tolerance, 54 lines defective in 49 genes had a chilling-sensitive phenotype. Of these genes, 16 encode proteins with plastid localization, of which four are plastid ribonucleoproteins (RNPs) (Wang et al., 2016). Three of the 16 – ORRM1, CP29A and CP31A – were previously characterized. CP31A and CP29A (for 31-kD and 29-kD chloroplast protein, respectively) are required for the stability of various mRNAs at low temperatures, and under these conditions they promote specific processing steps (Kupsch et al., 2012). The organelle RNA recognition motif (RRM) protein 1 (ORRM1) is an essential plastid editing factor in *A. thaliana* and maize (Sun et al., 2013). The newly identified RRM/RBD/RNP protein RBD1 binds directly to 23S rRNA, and more strongly under chilling conditions than at normal growth temperatures. Accordingly, the *rbd1* defect in chloroplast protein synthesis is particularly severe at low temperatures (Wang et al., 2016). Furthermore, the CRM (chloroplast RNA splicing and ribosome maturation) family member subfamily4 CFM4 (Lee et al., 2014) and the DEAD-box RNA helicase RH3 (Gu et al., 2014) play a positive role in seed germination and seedling growth under salt or cold stress conditions, because seed germination and seedling growth of the respective mutants are retarded under those conditions.

## Conclusion

The importance of OGE in stress acclimation responses has become increasingly apparent in recent years. Perturbations in OGE homeostasis trigger abiotic acclimation and tolerance responses, presumably via retrograde signaling. Thus, further studies on the molecular and physiological functions of OGE proteins should elucidate their roles in such responses. Moreover, as changes in proteins responsive to stress do not always reflect changes at the transcript level, posttranscriptional and translational mechanisms must be given more attention. Cell-fractionation experiments, together with metabolomics studies and the application of next-generation sequencing technologies like mRNA-Seq, global run-on (GRO)-Seq and global ribosomal profiling (Ribo-Seq), should ultimately allow us to assemble an integrated picture of how environmental changes regulate sub-cellular states and reveal the extent and nature of retrograde signal transduction.

## Author Contributions

TK drafted the manuscript, LW prepared the figure and the table, DL and TK finalized the manuscript.

## Conflict of Interest Statement

The authors declare that the research was conducted in the absence of any commercial or financial relationships that could be construed as a potential conflict of interest.
